# Shared and Distinct Gut Microbial Profiles in Saudi Women with Metabolically Healthy and Unhealthy Obesity

**DOI:** 10.3390/microorganisms11061430

**Published:** 2023-05-29

**Authors:** Ghadeer S. Aljuraiban, Mohammad A. Alfhili, Madhawi M. Aldhwayan, Esra’a A. Aljazairy, Sara Al-Musharaf

**Affiliations:** 1 Department of Community Health Sciences, College of Applied Medical Sciences, King Saud University, Riyadh 11451, Saudi Arabia; maldhwayan@ksu.edu.sa (M.M.A.); jz.esraa@gmail.com (E.A.A.); salmosharruf@ksu.edu.sa (S.A.-M.); 2 Department of Clinical Laboratory Sciences, College of Applied Medical Sciences, King Saud University, Riyadh 11451, Saudi Arabia; malfeehily@ksu.edu.sa

**Keywords:** obesity, obesity phenotype, gut microbiota, gut flora

## Abstract

Background: Mounting evidence suggests a pivotal role for the gut microbiome in energy disequilibrium characteristic of obesity. The clinical utility of microbial profiling for the distinction between metabolically healthy obesity (MHO) and metabolically unhealthy obesity (MUO) remains ill-defined. We aim to probe microbial composition and diversity in young adult Saudi females with MHO and MUO. This observational study included anthropometric and biochemical measurements and shotgun sequencing of stool DNA for 92 subjects. α- and β-diversity metrics were calculated to determine the richness and variability in microbial communities, respectively. Results showed that *Bacteroides* and *Bifidobacterium merycicum* were less abundant in MUO compared to healthy and MHO groups. BMI was negatively correlated with *B. adolescentis*, *B. longum*, and *Actinobacteria* in MHO, while being positively correlated with *Bacteroides thetaiotaomicron* in both MHO and MUO. Positive correlations between waist circumference and *B. merycicum* and *B. thetaiotaomicron* were observed in MHO and MUO, respectively. Compared to MHO and MUO groups, higher α-diversity was detected in healthy individuals who also had higher β-diversity compared to those with MHO. We conclude that modulation of the gut microbiome cohorts through prebiotics, probiotics, and fecal microbiota transplantation may be a promising preventive and therapeutic approach to obesity-associated disease.

## 1. Introduction

Obesity is defined as the presence of high body fat that may be related to adverse health conditions. Body mass index (BMI), which is determined by a simple calculation of weight and height (weight (kg)/height (m^2^)), is used to define overweight (≥25) and obesity (≥30) [1].

Obesity has been shown to increase the risk of type 2 diabetes, dyslipidemia, hypertension, cardiovascular diseases, and cancers, with these factors often occurring in tandem [2]. However, at an individual level, the limitations inherent in using BMI as a tool, such as an inability to distinguish between fat and lean tissue and between fat storage sites, means it is not a diagnostic tool for assessing health status [3]. In fact, obesity can and does exist in metabolically healthy individuals, called metabolically healthy obesity (MHO) [4,5]. MHO can be defined as obesity without metabolic abnormalities [4]. People with MHO tend to have less systemic inflammation despite having a similar fat mass, and some studies have shown a decreased risk of mortality and disease among people with MHO compared to those with metabolically unhealthy obesity (MUO) [5,6].

The possible role of gut microbiota in determining why some people with obesity are metabolically healthy and others are not is an emergent area of study [7]. The gut microbiota, the collective term for the microbial community inhabiting the gastrointestinal tract, has been recognized for its role in maintaining metabolic health [8,9,10]. It performs crucial functions in the body, including the maintenance of metabolic homeostasis [8], the regulation of immunity [11], the strengthening of the intestinal epithelium [12], and protection against pathogens [13]. Maintaining homeostasis in the gut microbiota is critical, as altered bacterial composition, or dysbiosis, is an acknowledged factor in developing infections and inflammatory diseases [14], insulin resistance [15,16], and obesity [17,18]. While certain groups of microorganisms dominate the gut, amounts and species vary greatly [19]. Identifying microbial composition can help in efforts to modulate the gut microbiota to manage and prevent adverse health outcomes.

In 2020, Kim et al. carried out one of the first studies that investigated the differences in gut microbiota between metabolically healthy and unhealthy adults with overweight and obesity and found significant differences in bacterial composition and alpha diversity [7]. While these findings may help develop strategies to prevent the progression of metabolic abnormalities among those with obesity, the generalizability may be limited [7]. Differences in gut microbiota composition can be ascribed to the heterogeneity of groups [20], lifestyle habits [21], cultural and dietary traditions [22], genetic factors [20], or study techniques [21,23]. Extrapolating the results from one cohort or to generalized biological relevance may be inappropriate [20]. Thus, it is necessary to perform population and geography-specific studies to enhance our understanding of the relationship between gut microbiota and health [20,24]. This study aims to explore whether there are differences in gut microbiota in young adult females with MHO and MUO in Saudi Arabia.

## 2. Materials and Methods

### 2.1. Study Design

The present study is an analytical, case-control investigation that builds upon previously published methods [25]. Female college students over 18 years of age were recruited between January 2019 and March 2020. We used various recruitment methods such as emails, social media networks, etc. Those included were females with a BMI of ≥30 kg/m^2^ (obese) or BMI = 18.5–24.9 kg/m^2^ (normal weight).

Out of the initial 400 women who expressed interest, 290 met the eligibility criteria. Of those, we excluded 198 individuals for various reasons, e.g., those who had chronic illnesses such as acute/chronic diarrhea in the past two months, pregnant females, those on special diets (e.g., weight-reduction), those using multi-vitamins or antibiotics, or those who did not hand in stool samples. Ultimately, 92 females were part of the final sample, and they signed a written consent to participate in the study. The Institutional Ethics Committee at King Saud University (KSU-IRB #E-19-3625) reviewed and approved the study protocol.

### 2.2. Biochemical Data

Participants fasted for more than 10 h overnight before two 5-mL tubes of blood were collected at the study clinic. Serum was removed from the blood and centrifuged within minutes of collection. The serum was then sent to the study laboratory for analysis. A biochemical analyzer (Konelab, Espoo, Finland) measured fasting blood glucose (FBG) levels (mmol/L), and a LIAISON XL analyzer (DiaSorin, Saluggia, Italy) measured insulin levels (mU/L). The homeostasis model assessment of insulin resistance (HOMA-IR) index was determined using the following calculation: [fasting serum insulin × (fasting glucose)/22.5]. Lipid profiles were measured with a biochemical analyzer (Konelab, Espoo, Finland). Previously published equations were used to determine the levels of low- and high-density lipoprotein cholesterol (LDL-C and HDL-C, respectively) [26]. High-sensitivity C-reactive protein (hs-CRP) was assessed using commercial enzyme-linked immunosorbent assay kits.

### 2.3. Anthropometric Indices

Weight and height measurements to the nearest 0.1 kg and 0.1 cm, respectively, were taken twice, with participants wearing light clothing and no shoes. BMI was calculated as kilograms divided by the square of height in meters. Waist circumference was measured according to the World Health Organization standard at the lowest rib, umbilicus, and narrowest waist area [27]. Hip circumference was measured twice to the nearest 0.5 cm with an inelastic tape around the great trochanter with legs close together. A third measurement was taken if the first two differed by more than 2 cm, and then an average of the two most similar measurements was used. Body composition data were identified with bioelectrical impedance analysis (BIA) using an InBody 770 machine (Seoul, Republic of Korea) [28].

### 2.4. Obesity Phenotypes

Participants were categorized based on their BMI into the following groups: normal weight (BMI 18.5–24.9 kg/m^2^) and obesity (BMI ≥ 30 kg/m^2^). A waist circumference of ≥85 cm was classified as abdominal obesity [29]. Participants with HOMA-IR levels in the top quartile were considered insulin resistant [30].

MHO and MUO phenotypes were defined as per National Cholesterol Education Program-Adult Treatment Panel III (ATPIII) [31] and Karelis [32], based on obesity markers (BMI or waist circumference) and specific cardiometabolic abnormalities. Participants were classified as MHO if they presented with one of the obesity markers but did not have any metabolic abnormalities. Participants were categorized as MUO if they presented with obesity (either BMI- or waist circumference-defined) and three or more metabolic abnormalities [33], including: FBG, ≥5.6 mmol/L or on treatment, triglyceride ≥ 1.7 mmol/L or on treatment, HDL-C < 1.29 mmol/L, LDL-C ≤ 2.6 mmol/L, HOMA-IR level in the top quartile. Finally, participants were classified as ‘healthy’ if they had normal weight and none of the metabolic abnormalities.

### 2.5. Stool Collection Characterization of Gut Composition

Stool samples were gathered under sterile conditions using dry, screw-cap containers. The samples were then placed in a large container with dried ice and transported to the study lab. There, samples were promptly frozen at −80 °C until further processing. DNA extraction was executed using 0.25 g aliquots of frozen stool and the QIAamp Power Fecal DNA Isolation Kit (Qiagen, Hilden, Germany) in line with the manufacturer’s instructions. The DNA was eluted in a 100 μL C6 elution buffer. Its purity and concentration were assessed with a Nano Drop spectrophotometer (Nano Drop Technologies, Wilmington, DE, USA). The Illumina Nextera XT Library Preparation Kit (Illumina, Inc., San Diego, CA, USA) was used for DNA library preparation. Sequencing was conducted on an Illumina sequencer. For accurate quantification, a Qubit^®^ fluorometer was utilized.

Gut microbiota composition was determined with genomic sequencing using the WGS analysis method [34]. This approach enabled the identification of major phyla in the gut. The CosmosID bioinformatics platform (CosmosID, Inc., Germantown, MD, USA) was utilized to analyze the sequencing data. With this method, we were able to profile antibiotic resistance and virulence genes and quantify the relative abundance of organisms in the multi-kingdom microbiome [35,36,37,38]. High-performance data mining and the curated genome databases enabled the rapid identification of distinct sequences that produced microbes. In addition, the CosmosID antibiotic resistance and virulence-associated gene databases were used to search the unassembled sequence reads for the identification of the community resistome.

### 2.6. Dietary Data

We collected information on food and drink intake using the Saudi Food and Drug Authority Food Frequency Questionnaire (FFQ) [39], consisting of 133 food and beverage items. Dietitians who were experts in the field asked participants about their habitual food and drink intake, while estimating portion sizes using food modules. For nutrition analysis, a food processor software, ESHA version 11.1 (ESHA Research, Salem, OR, USA), was used.

### 2.7. Statistical Analyses

IBM SPSS Statistics for Windows (version 24; IBM Corp, Armonk, NY, USA) was used to conduct statistical analyses. Normality tests for quantitative variables were conducted prior to analysis. Means with standard deviations were used for continuous variables and frequencies were used for categorical variables. Means and standard deviations of anthropometric indices, biochemical data, and gut flora according to obesity phenotype; healthy, MHO, MUO, were compared with one-way analysis of variance (ANOVA). The correlation between metabolic markers and gut flora for each obesity phenotype was assessed with Pearson’s correlation coefficient. Statistical significance was set at a *p*-value of ≤0.05.

The α-diversity, a measure of gut microbiota richness, was determined using the CosmosID taxonomic analysis (R software Vegan package, version 2.5-6). The gut composition at the species-level relative abundance matrices was determined using β-diversity. Wilcoxon Rank-Sum tests and the ggsignif package for R were used to calculate the statistical difference between phenotypes for α-diversity by Shannon index. The nonparametric PERMANOVA analysis was conducted using Vegan’s function adonis2 based on the Jaccard index for β-diversity analysis.

## 3. Results

### 3.1. Characteristics of Participants

The current study included 92 females with a (mean ± SD) age of (21.1 ± 1.5 years) Table S1. Anthropometric data were as follows; BMI (28.56 ± 8.0 kg/m^2^), waist-to-hip ratio (0.7 ± 0.1), fat percentage (42.5 ± 9.4%), and muscle percentage (28.2 ± 7.0%). Total cholesterol was (4.1 ± 1.5 mmol/L), HDL-C (1.0 ± 0.3 mmol/L), LDL-C (2.9 ± 1.3), triglycerides [0.7 (0.5–1.0) mmol/L], FBG (4.6 ± 0.7 mmol/L), insulin (9.9 ± 11.2 µIU/mL), HOMA-IR (2.0 ± 2.8) and hs-CRP (1.5 ± 1.0 mg/L).

### 3.2. Anthropometric Indices, Biochemical Data, and Gut Flora Stratified by Obesity Phenotype

When participants were divided according to obesity phenotype, markers of obesity such as BMI and waist were higher in MHO (*n* = 19) and MUO (*n* = 19) compared to healthy individuals (*n* = 48) (Table 1). Total cholesterol, LDL, triglycerides, fasting blood sugar, insulin, HOMA-IR, and hs-CRP were higher in MUO compared to those with normal weight (*p* < 0.01). Those with MUO had lower abundance of *Bacteroides (unidentified species)* (*p* < 0.06), *Bacteroides uniformis* and *Bifidobacterium merycicum* (*p* < 0.03) compared to healthy individuals and MHO. For dietary intake, total calorie intake was higher in MUO compared to MHO and healthy individuals, however the difference was not significant. Total fat intake (%) was higher in MUO than MHO and healthy individuals (*p* < 0.05) (Table 1).

### 3.3. Correlations of Metabolic Markers with Gut Flora for Each Obesity Phenotype

Among healthy individuals, there was a positive correlation between BMI and *Bifidobacterium kashiwanohense* (r = 0.24) (Table 2). *Flavonifractor plautii* was positively correlated with two markers; HDL-C and (r = 0.49) and triglycerides (r = 0.39).

*Bifidobacterium longum* was inversely correlated with waist (r = −0.25) and hs-CRP (r = −0.24). *Proteobacteria* was also inversely correlated with several metabolic markers; triglycerides (r = −0.27), insulin (r = −0.30), and HOMA-IR (r = −0.30) (Table 2).

Table 3 presents the correlation between metabolic markers and gut flora in the MHO phenotype. *Bacteria_u_p* was inversely correlated with both BMI (r = −0.45) and insulin (r = −0.48). Similarly, *Bifidobacterium adolescentis* was inversely correlated with a number of metabolic markers; BMI (r = −0.54), HDL-C (r = −0.46), FBG (r = −0.55), insulin (r = −0.61), and HOMA-IR (r = −0.66). *Bifidobacterium longum* was also inversely correlated with BMI (r = −0.43), FBG (r = −0.54), and HOMA-IR (r = −0.42).

*Bifidobacterium merycicum* was positively correlated with waist (r = 0.47) and inversely with a number of metabolic markers including BMI (r = −0.26), triglycerides (r = −41), insulin (r = −0.46), and HOMA-IR (r = −0.43).

*Actinobacteria* was inversely correlated with several markers; BMI (r = −0.49), FBG (r = −0.51), insulin (r = −0.46), and HOMA-IR (r = −0.54).

Positive correlations were observed between *Akkermansia muciniphila* and both FBG (r = 0.45) and HOMA-IR (r = 0.51). Similarly, *Verrucomicrobia* showed positive correlations with insulin (r = 0.42) and HOMA-IR (0.52). Further, *Bacteroides faecichinchillae* was positively correlated with BMI and hs-CRP (r = 0.44, 0.46, respectively) (Table 3).

In the MUO phenotype, *Clostridium difficile* was positively correlated with several metabolic markers; BMI and waist (r = 0.42, 0.47, respectively) and inversely with HDL-C (r = −0.50). *Bacteroides faecichinchillae* was positively correlated a number of markers; BMI (r = 0.71), waist (r = 0.68), and HOMA-IR (r = 0.40).

HDL-C was positively correlated with *Akkermansia muciniphila* (r = 0.44), *Verrucomicrobia* (r = 0.44), *Bacteroides faecichinchillae* (r = 0.50) and inversely with *Clostridium bolteae* (r = −0.50) (Table 4).

### 3.4. α- and β-Diversity in Each Obesity Phenotype

**Figure 1:**
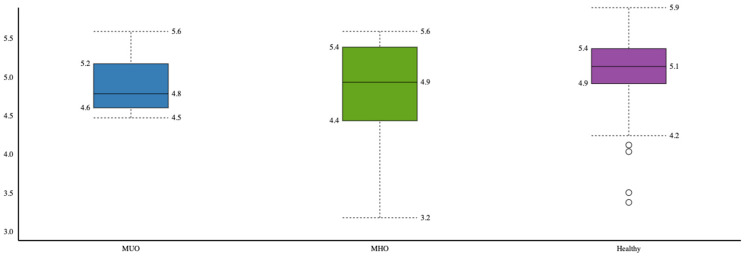
Comparison of microbial α-diversity between obesity phenotypes (healthy, MHO and MUO).

Healthy individuals had higher microbial α-diversity based on the Shannon index compared to the MHO and MUO phenotypes, and the difference was marginally significant (*p* = 0.06) (Figure 1).

**Figure 2:**
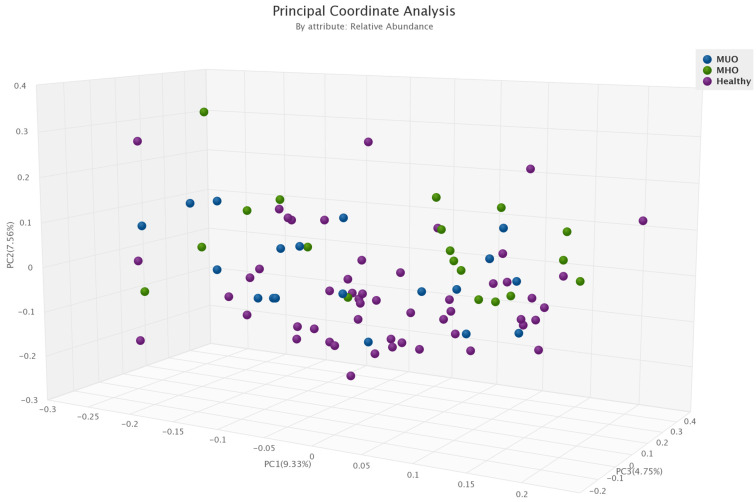
Comparison of microbial β-diversity between obesity phenotypes (healthy, MHO and MUO).

The overall microbial β-diversity was significant between the three groups MHO, MUO and the healthy group (*p* = 0.01) (Figure 2). Among obesity phenotypes, healthy individuals had higher β-diversity compared to MHO (*p* = 0.005).

## 4. Discussion

Outcomes from this study add to the mounting body of literature demonstrating differences in the gut microbiota between people with MUO and MHO. This work investigated the variations in gut microbiota among metabolically healthy and metabolically unhealthy young females with obesity in Saudi Arabia.

The main findings reveal that those with MUO have a lower abundance of *Bacteroides, Bacteroides uniformis*, and *Bifidobacterium merycicum* compared to both healthy individuals without obesity and compared to those with MHO. These bacteria have essential functions in metabolic health, which may explain these distinctions. *Bacteroides* is a genus of anaerobic, non-spore-forming, bile-resistant, gram-negative rods and includes over 30 species of bacteria [40]. Of all gram-negative bacteria, it is the most abundant one colonizing the human gastrointestinal tract [40]. *Bacteroides* has been demonstrated to enhance host immunity, conserve microecological balance in the gut, and speed up angiogenesis in the intestinal mucosa [40]. Moreover, some research has shown that more *Bacteroides* in the gastrointestinal tract could reduce obesity risk [41,42]. *Bacteroides uniformis*, a type of *Bacteroides*, exhibits significant glycolytic capability, and it can adapt to various gut environments [43]. Low levels of *Bacteroides uniformis* have been associated with obesity risk [44] and, by potentially limiting the biosynthesis of lipopolysaccharides, which promote pro-inflammatory cytokines, *Bacteroides uniformis* could help alleviate inflammation [43]. *Bifidobacterium merycicum* belongs to the *Bifidobacterium genus*, which has been positively associated with host health, including demonstrations of preventing and/or treating colorectal cancer [45], diarrhea [46], inflammatory bowel disease [47] and obesity [44,48,49].

We also found that the Shannon index, which indicated microbial alpha diversity (richness), was lower in the MUO group than in the MHO group. The microbial β-diversity significantly differed between all three groups (MUO, MHO, and healthy without obesity). Our prior research in this same cohort investigating the difference in gut microbiota between participants with and without obesity (regardless of phenotype) likewise found that alpha diversity was not associated with BMI [25]. However, those with obesity had a distinct gut microbiota profile compared to those without obesity [25]. The literature has also previously established the relationship between various metabolic factors and microbial diversity. For instance, lower microbial richness has been associated with unfavorable lipid profiles (i.e., high triglycerides and low HDL) [50], and a higher Shannon index and richness have been associated with lower rates of insulin resistance [51].

Limited research has investigated the difference in gut microbiota between MHO and MUO groups. Much of the work that has been done has found similar results to the present study. Kim et al. found that alpha diversity was lower among the MUO than both the MHO and the healthy non-obesity group in their study of 747 adults with overweight or obesity [7]. The microbial composition also differed between the three study groups, and they noted differences in bacterial composition between those with MUO and MHO [7]. A comparable research question has also been investigated among children and older adults with obesity [52,53]. Children with MUO had a decreased alpha diversity and richness compared to those with MHO; however, microbial β-diversity did not differ [52]. On the other hand, there was neither alpha nor β-diversity differed between the MHO and MUO groups among older adults [53]. The present study and the previous studies have all emerged with variations in the bacterial species that stood out as significantly different between the groups [7,52,53,54]. Such an outcome was anticipated considering different cohorts, age groups, and methods were used for each study, and these are key aspects impacting the gut microbiota or the measurement of it [7,52,53,54,55,56].

Another area of study that future efforts can focus on is microbial metabolic potential. Gut microbes consume, exchange, and secrete metabolites, which are small molecules created from metabolic activity [57]. Metabolites enter the human gut through the intake of nutrients and are consumed by microbes which then convert some of the metabolites into their biomass and secrete the rest for consumption by other microbes [57]. Eventually, the remaining metabolites will exit the gastrointestinal tract [57]. Microbes interact through these metabolites to maintain homeostasis in the gastrointestinal tract, but metabolites can also impact the host [58,59,60]. In fact, some research indicates that host health is more related to metabolic pathways than microbe taxonomy [61,62]. Visconti et al. found that metabolic pathways are more likely to be shared between people than species are, sharing an average of 82% compared to just 43%, respectively [61]. Specifically, metabolic pathways were associated with 95% of fecal and 34% of blood metabolites compared to the microbiome, which was associated with 71% and 15%, respectively [61]. Thus, some of the discrepancies in research focusing solely on the taxonomy of the gut microbiota may be further explained by exploring the metabolome as well. Metabolites have also been studied in terms of MUO and MHO, demonstrating key differences. A recent systematic review of studies investigating the difference in metabolomic signature between MUO and MHO found that the overall signature for MHO trended more favorably [63]. Specifically, amino acids (branched-chain and aromatic), lipids, and acylcarnitines may be elevated in MUO [63].

One consideration when interpreting these results is the theory that MHO is a steppingstone to MUO. Previous work has shown that between 33% and 48% of people with MHO end up with MUO within 5 to 10 years, which may explain why MHO is less common in older age groups [64]. As such, longitudinal studies examining how the gut microbiota changes over time and the potential for modulating gut microbiota to avoid or delay such a progression may be useful.

The present study also demonstrated a higher hs-CRP level among those with MUO than those with MHO, both of which had higher levels than participants that were metabolically healthy without obesity. Previous work has come to conflicting conclusions in this regard, with some studies reporting higher hs-CRP levels among MUO compared to MHO [65,66], some finding similar levels among obesity phenotypes [67,68,69], and others finding higher levels among those with obesity in general compared to metabolically healthy individuals without obesity [66,67,68,69] or metabolically unhealthy individuals without obesity [69]. Variations in findings may result from the cohorts used as well as the statistical treatments (e.g., adjusting for abdominal obesity or body fat percentage) [67].

The present study had several strengths. Data collection accuracy was enhanced through the collection of information through several channels, including in-person interviews, in-depth health history questionnaires, and repeated measures of anthropometric data. This study used whole-genome sequencing, which has heightened accuracy, can detect more microbial species, and has better microbial resolution than the commonly used 16S rRNA sequencing [70]. In terms of limitations, the cohort in this study was exclusive of people with overweight and included only those with normal weight or obesity. While this limits the interpretation that can be made with regard to the gut microbiota of those with overweight, it more distinctly categorizes individuals into the extreme BMI groups for comparisons. As an observational study, causality cannot be confirmed, and confounding cannot be rejected. Lastly, while the selection of a specific cohort of young females in Saudi Arabia addresses the lack of research previously conducted in this population, the generalizability to other groups is limited, which is a drawback common to gut microbiota studies [20,24].

## 5. Conclusions

In conclusion, this study was the first to examine differences in gut microbiota among young females with MHO and MUO in Saudi Arabia. The findings support work demonstrating differences in diversity and microbial composition between phenotypes of obesity. Further investigations are required to identify the mechanisms underlying the relationship between the microbiome and metabolism in MHO and MUO, which may play a key part in preventing metabolic disease. Modulation of the gut microbiome cohorts through prebiotics, probiotics, and fecal microbiota transplantation may be a promising preventive and therapeutic approach to obesity-associated disease.

## Figures and Tables

**Table 1:** Anthropometric indices, biochemical data, and gut flora by obesity phenotype, *n* = 92 ^1^.

Variables	Healthy (*n* = 48)	Metabolically Healthy Obesity (*n* = 19)	Metabolically Unhealthy Obesity (*n* = 19)	*p*-Value
Age (years)	21.1 (21.1–21.1)	21.1 (21.1–21.1)	21.1 (21.1–21.1)	1.00
Energy (Kcal/day)	4397.3 (2871.2–5923.5)	3078.9 (546.8–5611)	3340.9 (680.6–6001.2)	0.61
BMI (kg/m^2^)	23.3 (22.1–24.5)	36.2 (34.2–38.2)	35.8 (33.7–38)	<0.0001
Waist (cm)	68 (65.6–70.3)	97.8 (94–101.7)	99.1 (95.1–103.1)	<0.0001
WHR (ratio)	0.7 (0.7–0.7)	0.8 (0.8–0.9)	0.8 (0.8–0.9)	<0.0001
Fat (%)	38.1 (36–40.3)	53.1 (50.1–56)	51.2 (48.3–54)	<0.0001
Muscle mass (%)	29.5 (27.1–31.8)	26.3 (22.5–30.2)	27.2 (23.2–31.3)	0.33
Biochemical Data				
Total Cholesterol (mmol/L)	3.7 (3.3–4.1)	3.8 (3.1–4.5)	5.2 (4.5–5.8)	0.002
HDL-C (mmol/L)	1 (0.8–1.1)	1.1 (0.9–1.2)	1.1 (0.9–1.2)	0.41
LDL-C (mmol/L)	2.7 (2.3–3)	2.6 (2–3.2)	3.9 (3.4–4.5)	0.001
Triglycerides (mmol/L)	0.6 (0.5–0.7)	1 (0.8–1.1)	1 (0.9–1.2)	<0.0001
FBG (mmol/L)	4.3 (4.1–4.5)	5 (4.6–5.3)	4.8 (4.4–5.2)	0.01
Insulin (µIU/mL)	7.9 (6.4–9.4)	15.7 (13.3–18.2)	17.4 (14.9–19.9)	<0.0001
HOMA-IR	1.4 (1.1–1.8)	3.5 (2.9–4.1)	3.7 (3.2–4.3)	<0.0001
hs-CRP (mg/L)	2.2 (7.6–3.7)	5.9 (3.5–8.3)	10.4 (8.3–13.2)	<0.0001
Gut flora				
*Bacteroidetes*	0.7 (0.7–0.7)	0.7 (0.6–0.8)	0.8 (0.7–0.8)	0.23
*Bacteria (unidentified phylum)*	0.001 (0.001–0.001)	0.001 (0.0002–0.001)	0.0004 (−0.0003–0.001)	0.41
*Bacteroides (unidentified species)*	0.003 (0.001–0.005)	0.01 (0.003–0.01)	0.01 (0.003–0.01)	0.06
*Bacteroides uniformis*	0.1 (0.1–0.1)	0.1 (0.03–0.1)	0.1 (0.1–0.1)	0.03
*Bifidobacterium adolescentis*	0.01 (0.01–0.01)	0.01 (0.002–0.01)	0.01 (−0.0003–0.01)	0.51
*Bifidobacterium kashiwanohense*	0.001 (0.001–0.002)	0.001 (0.0002–0.002)	0.0005 (−0.0005–0.001)	0.51
*Bifidobacterium longum*	0.01 (0.01–0.01)	0.01 (0.003–0.01)	0.01 (0.003–0.01)	0.69
*Bifidobacterium merycicum*	0.00002 (−0.0001–0.0001)	0.0003 (0.0001–0.0004)	0.00001 (0.0002–0.0002)	0.03
*Clostridium difficile*	0.0001 (−0.00003–0.0002)	0.00004 (−0.0002–0.0003)	0.0002 (−0.00004–0.0004)	0.63
*Clostridium Bolteae*	0.001 (0.0003–0.001)	0.001 (0.0002–0.002)	0.001 (−0.0001–0.002)	0.82
Fusobacteria	0.00005 (−0.0002–0.0003)	0.00001 (−0.0004–0.0004)	0.0003 (−0.0001–0.001)	0.43
*Actinobacteria*	0.04 (0.03–0.1)	0.04 (0.02–0.1)	0.03 (0.01–0.04)	0.18
*Akkermansia muciniphila*	0.01 (0.003–0.01)	0.002 (−0.003–0.01)	0.003 (−0.002–0.01)	0.28
*Proteobacteria*	0.01 (0.01–0.02)	0.02 (0.01–0.02)	0.01 (0.01–0.02)	0.73
*Faecalibacterium Prausnitzii*	0.02 (0.02–0.03)	0.02 (0.02–0.03)	0.02 (0.01–0.03)	0.76
*Firmicutes*	0.2 (0.2–0.3)	0.2 (0.2–0.3)	0.2 (0.2–0.3)	0.39
*Flavonifractor plautii*	0.001 (0.001–0.001)	0.001 (0.0005–0.002)	0.001 (0.0004–0.002)	0.98
*Lactobacillus acidophilus*	0.00002 (−0.00003–0.0001)	0.0001 (0.0001–0.0002)	0.00001 (−0.0001–0.0001)	0.32
*Verrucomicrobia*	0.01 (0.003–0.01)	0.002 (−0.003–0.01)	0.003 (−0.002–0.01)	0.31
Dietary Data				
Total energy (kcal/d)	3222 (841–1503)	3149 (678–1619)	4564 (1951–2177)	0.08
Carbohydrate (%)	49.3 (44.0–54.5)	41.7 (36.0–47.4)	45.7 (42.0–49.4)	0.15
Protein (%)	16.3 (13.5–18.2)	15.2 (13.2–17.2)	15.5 (14.2–16.8)	0.60
Fat (%)	34.4 (29.5–39.2)	38.7 (35.3–42.2)	43.1 (37.9–48.4)	0.05

^1^ Data are presented as mean (95% CI) as analyzed by ANOVA; Body mass index (BMI), fasting blood glucose (FBG), high-density lipoprotein cholesterol (HDL-C), high-sensitivity C-reactive protein (hs-CRP), homeostatic model assessment for insulin resistance (HOMA-IR), low-density lipoprotein cholesterol (LDL-C), Waist-to-hip ratio (WHR).

**Table 2:** Correlation coefficients between metabolic markers and gut flora in healthy individuals, *n* = 48.

	BMI	Waist	HDL-C (mmol/L)	LDL-C (mmol/L)	TG (mmol/L)	FBG (mmol/L)	Insulin (µIU/mL)	HOMA-IR	hs-CRP ng/mL
*Bacteroidetes*	−0.07	0.16	−0.13	−0.04	−0.04	0.03	0.23	0.23	−0.11
*Bacteria (unidentified phylum)*	0.07	0.03	0.02	−0.01	0.24	0.09	−0.09	−0.10	0.01
*Bacteroides (unidentified species)*	−0.16	0.07	0.16	−0.16	−0.12	0.01	−0.01	0.04	−0.22
*Bacteroides uniformis*	0.24 *	−0.08	0.09	−0.05	−0.02	−0.16	0.08	−0.11	−0.01
*Bifidobacterium adolescentis*	−0.06	0.05	0.02	0.01	−0.09	−0.03	−0.14	−0.10	−0.26 *
*Bifidobacterium kashiwanohense*	−0.11	0.01	0.10	0.05	0.12	−0.19	−0.22	−0.21	−0.01
*Bifidobacterium longum*	−0.01	−0.25 *	0.07	0.03	0.01	−0.10	−0.13	−0.20	−0.24 *
*Bifidobacterium merycicum*	−0.01	−0.01	−0.20	0.06	−0.01	−0.26 *	0.02	−0.05	−0.11
*Clostridium difficile*	−0.15	−0.08	−0.08	−0.02	−0.05	−0.05	0.09	0.05	−0.11
*Clostridium Bolteae*	0.14	0.09	0.22	0.01	0.13	0.22	−0.18	−0.17	−0.02
*Actinobacteria*	−0.06	−0.08	0.19	0.07	0.05	−0.09	−0.25 *	−0.22	−0.14
*Akkermansia muciniphila*	−0.11	−0.02	0.01	0.13	0.05	−0.02	−0.16	−0.13	−0.02
*Proteobacteria*	−0.07	−0.02	−0.09	−0.24	−0.27 *	−0.18	−0.30 *	−0.30 *	−0.20
*Faecalibacterium Prausnitzii*	0.07	−0.09	0.03	0.09	0.03	0.06	0.01	0.06	0.27
*Firmicutes*	0.13	−0.17	0.11	0.04	0.05	0.01	−0.14	−0.17	0.20
*Flavonifractor plautii*	0.17	0.16	0.49 *	0.04	0.39 *	0.15	−0.07	−0.06	0.10
*Lactobacillus acidophilus*	0.11	−0.20	0.01	−0.20	−0.17	−0.16	0.09	−0.14	−0.06
*Verrucomicrobia*	−0.10	−0.02	0.01	0.10	0.03	−0.03	−0.16	−0.13	−0.01
*Bacteroides faecichinchillae*	−0.14	−0.05	−0.05	−0.23	−0.21	−0.25	0.03	−0.07	−0.10
*Bacteroides thetaiotaomicron*	−0.01	−0.04	0.07	0.24	0.17	0.19	−0.04	0.04	−0.21
*Bifidobacterium pseudocatenulatu*	−0.11	−0.12	0.07	0.14	0.16	0.01	−0.14	−0.08	0.01

* indicates *p* < 0.05. Body mass index (BMI), fasting blood glucose (FBG), high-density lipoprotein cholesterol (HDL-C), high-sensitivity C-reactive protein (hs-CRP), homeostatic model assessment for insulin resistance (HOMA-IR), low-density lipoprotein cholesterol (LDL-C), triglycerides (TG), waist-to-hip ratio (WHR).

**Table 3:** Correlation coefficients between metabolic markers and gut flora in individuals with metabolically healthy obesity, *n* = 21.

	BMI	Waist	HDL-C (mmol/L)	LDL-C (mmol/L)	TG (mmol/L)	FBG (mmol/L)	Insulin (µIU/mL)	HOMA-IR	hs-CRP ng/mL
*Bacteroidetes*	0.31	0.19	0.30	−0.02	0.20	0.24	0.25	0.28	0.07
*Bacteria (unidentified phylum)*	−0.45 *	−0.11	−0.02	−0.06	0.10	0.15	−0.48 *	−0.37	−0.23
*Bacteroides (unidentified species)*	−0.12	−0.26	0.10	−0.01	0.79 *	0.13	0.17	0.18	−0.30
*Bacteroides uniformis*	−0.04	−0.04	−0.06	−0.07	−0.32	−0.29	−0.25	−0.28	−0.04
*Bifidobacterium adolescentis*	−0.54 *	−0.24	−0.46 *	−0.08	−0.28	−0.55 *	−0.61 *	−0.66 *	−0.40
*Bifidobacterium kashiwanohense*	−0.21	−0.08	−0.04	0.07	−0.10	−0.48 *	−0.13	−0.25	0.38
*Bifidobacterium longum*	−0.43 *	−0.07	−0.40	−0.03	−0.20	−0.54 *	−0.33	−0.42 *	−0.15
*Bifidobacterium merycicum*	−0.26	0.47 *	−0.02	−0.05	−0.41 *	−0.14	−0.46 *	−0.43 *	−0.12
*Clostridium difficile*	−0.23	−0.25	0.23	−0.02	0.79 *	0.08	0.10	0.10	−0.20
*Clostridium Bolteae*	0.13	0.16	0.36	0.29	0.02	−0.03	0.25	0.20	0.46
*Actinobacteria*	−0.49 *	−0.02	−0.32	−0.10	−0.28	−0.51 *	−0.46 *	−0.54 *	0.03
*Akkermansia muciniphila*	0.24	−0.04	−0.12	−0.09	−0.32	0.45 *	0.41	0.51 *	0.04
*Proteobacteria*	−0.32	−0.33	−0.42	−0.13	−0.17	−0.65 *	−0.21	−0.35	−0.30
*Faecalibacterium Prausnitzii*	−0.17	−0.38	0.17	0.14	0.61 *	−0.06	0.11	0.08	−0.29
*Firmicutes*	−0.16	−0.16	−0.19	0.08	−0.12	−0.03	−0.13	−0.13	−0.05
*Flavonifractor plautii*	0.07	0.20	0.35	0.29	−0.01	−0.01	0.17	0.13	0.38
*Lactobacillus acidophilus*	0.10	−0.02	0.28	0.13	−0.07	0.14	−0.02	0.02	0.46 *
*Verrucomicrobia*	0.24	−0.04	−0.12	−0.10	−0.30	0.46	0.42 *	0.52 *	0.05
*Bacteroides faecichinchillae*	0.44 *	0.18	0.09	0.05	−0.33	0.22	0.33	0.35	0.46 *
*Bacteroides thetaiotaomicron*	0.11	0.02	0.27	0.15	−0.05	0.07	−0.01	0.01	0.45 *

* indicates *p* < 0.05. Body mass index (BMI), fasting blood glucose (FBG), high-density lipoprotein cholesterol (HDL-C), high-sensitivity C-reactive protein (hs-CRP), homeostatic model assessment for insulin resistance (HOMA-IR), low-density lipoprotein cholesterol (LDL-C), triglycerides (TG), waist-to-hip ratio (WHR).

**Table 4:** Correlation coefficients between metabolic markers and gut flora in individuals with metabolically unhealthy obesity, *n* = 23.

	BMI	Waist	HDL-C (mmol/L)	LDL-C (mmol/L)	TG (mmol/L)	FBG (mmol/L)	Insulin (µIU/mL)	HOMA-IR	hs-CRP ng/mL
*Bacteroidetes*	0.16	0.24	−0.26	0.20	−0.03	0.05	0.08	0.11	−0.01
*Bacteria (unidentified phylum)*	0.36	0.32	−0.01	0.14	−0.07	0.12	0.26	0.31	0.31
*Bacteroides (unidentified species)*	0.26	0.33	−0.03	0.30	−0.14	0.24	0.09	0.22	0.24
*Bacteroides uniformis*	0.12	0.36	−0.02	0.01	−0.30	−0.03	0.18	0.21	0.31
*Bifidobacterium adolescentis*	−0.33	−0.28	0.10	−0.01	−0.15	−0.11	0.02	−0.02	−0.08
*Bifidobacterium kashiwanohense*	−0.03	−0.01	−0.12	−0.19	−0.40 *	−0.20	−0.19	−0.20	0.15
*Bifidobacterium longum*	−0.13	0.03	−0.21	−0.13	−0.33	−0.09	0.03	0.01	−0.07
*Bifidobacterium merycicum*	0.02	0.20	−0.29	−0.33	−0.24	0.11	−0.17	−0.14	−0.37
*Clostridium difficile*	0.42 *	0.47 *	−0.50 *	0.10	0.24	0.01	0.19	0.17	−0.01
*Clostridium Bolteae*	0.35	0.28	−0.33	0.38	0.55 *	−0.10	0.18	0.13	0.17
*Actinobacteria*	−0.27	−0.20	0.07	−0.20	−0.49 *	−0.30	−0.08	−0.14	0.03
*Akkermansia muciniphila*	−0.04	−0.20	0.44 *	−0.30	−0.32	−0.12	−0.02	−0.04	−0.18
*Proteobacteria*	0.05	−0.01	0.17	0.08	0.29	−0.20	−0.11	−0.16	0.25
*Faecalibacterium Prausnitzii*	−0.36	−0.48	−0.08	0.13	−0.09	0.17	−0.44 *	−0.37	0.06
*Firmicutes*	−0.13	−0.21	0.22	−0.17	0.12	0.03	−0.06	−0.07	−0.01
*Flavonifractor plautii*	0.47	0.45	0.09	0.02	0.10	0.19	0.34	0.39	−0.08
*Lactobacillus acidophilus*	−0.04	−0.20	0.44 *	−0.30	−0.32	−0.12	−0.02	−0.04	−0.18
*Verrucomicrobia*	−0.03	−0.13	0.50 *	−0.22	−0.08	0.17	0.07	0.10	−0.31
*Bacteroides faecichinchillae*	0.71 *	0.68 *	−0.06	−0.01	0.10	0.09	0.39	0.40 *	0.04
*Bacteroides thetaiotaomicron*	0.09	0.01	−0.07	−0.05	−0.25	−0.07	−0.09	−0.08	0.15

* indicates *p* < 0.05. Body mass index (BMI), fasting blood glucose (FBG), high-density lipoprotein cholesterol (HDL-C), high-sensitivity C-reactive protein (hs-CRP), homeostatic model assessment for insulin resistance (HOMA-IR), low-density lipoprotein cholesterol (LDL-C), triglycerides (TG), waist-to-hip ratio (WHR).

## Data Availability

Datasets generated for this study can be found in the Figshare repository: 10.6084/m9.figshare.20106176.

## Supplementary Materials

The following supporting information can be downloaded at: https://www.mdpi.com/article/10.3390/microorganisms11061430/s1, Table S1: General characteristics of total participants.
