# Supplementary material for: Shared and Distinct Gut Microbial Profiles in Saudi Women with Metabolically Healthy and Unhealthy Obesity
Source: Microorganisms. 2023 May 29;11(6):1430. doi: 10.3390/microorganisms11061430 (PMC10300982; doi:10.3390/microorganisms11061430)
Supplement: Supplementary file 1 [file microorganisms-11-01430-s001.zip › microorganisms-2399661-SI.pdf]

Supplementary File

Table S1. General characteristics of total participants[1]

Characteristics	Total (n=92)
Age (Years)	21.1 ± 1.5
Age of Menarche	12.4 ± 1.0
Energy (kcal/d)	3071.7 (2353.3 - 4106.3)
**Anthropometric measurements**	
Height (cm)	157.7 ± 5.2
Weight (kg)	70.7 ± 19.7
BMI (kg/m^2)	28.6 ± 8.0
Waist (cm)	80.4 ± 17.4
Hip (cm)	109.2 ± 16.4
WHR (ratio)	0.7 ± 0.1
Fat (%)	42.5 ± 9.4
Protein (kg)	7.6 ± 1.1
Skeletal muscle mass (kg)	21.0 ± 3.5
Muscle mass (%)	28.2 ± 7.0
Total Body Water (L)	28.7 ± 4.3
Fluid (%)	42.1 ± 6.8
**Biochemical data**	
Total Cholesterol (mmol/l)	4.1 ± 1.5
HDL-C (mmol/l)	1.0 ± 0.3
LDL-C (mmol/l)	2.9 ± 1.3
Triglyceride (mmol/l) #	0.7 (0.5 - 1.0)
FBG (mmol/l)	4.6 ± 0.7
Insulin (µIU/mL) #	9.9 (6.0 - 15.8)
HOMA-IR #	2.0 (1.1 - 3.6)
hs-CRP (ng/ml)	1.5 (7.9 – 7.6)

[1]Variables are presented as (mean ± standard deviation (SD)). Non-normal variables presented as median (1st quartile – 3rd quartile).

# Indicates non-normal variables.

Body mass index (BMI), fasting blood glucose (FBG), high-density lipoprotein cholesterol (HDL-C), high-sensitivity C-reactive protein (hs-CRP), homeostatic model assessment for insulin resistance (HOMA-IR), low-density lipoprotein cholesterol (LDL-C), Waist-to-hip ratio (WHR)
